# Editorial: Advancements in therapeutic strategies for skeletal muscle and cardiovascular diseases: integrating innovative approaches for enhanced outcomes

**DOI:** 10.3389/fphar.2026.1779437

**Published:** 2026-01-21

**Authors:** Sara Carolina Vieira Terra, Leonardo Martin, Roberta Sessa Stilhano

**Affiliations:** 1 Department of Physiological Sciences, Santa Casa de São Paulo School of Medical Sciences, SãoPaulo, Brazil; 2 Department of Pharmaceutical Sciences, University of Antwerp, Antwerp, Belgium; 3 Infla-Med Centre of Excellence, University of Antwerp, Antwerp, Belgium

**Keywords:** cardiovascular diseases, cell therapy, gene therapy, skeletal muscle, stem cells

Skeletal muscle disorders and cardiovascular diseases remain leading causes of disability and mortality worldwide, affecting individuals across diverse populations. Despite significant advances in conventional pharmacological, surgical, and rehabilitation-based approaches, many patients continue to face incomplete recovery, disease progression, or long-term functional impairment. These limitations reflect the biological complexity of muscle and cardiovascular tissues and highlight the need for therapeutic strategies that extend beyond symptom control toward restoring or reprogramming diseased cells, aligning with the emerging field of regenerative pharmacology and advanced therapeutic medicinal products.

In this context, cell and gene therapies are reshaping the therapeutic landscape. Recent regulatory approvals of advanced therapy products mark a critical inflection point, demonstrating that these approaches can be successfully translated from experimental platforms into clinically validated interventions. Collectively, this momentum supports a shift toward more durable, mechanism-driven treatments and sets the stage for new opportunities to improve outcomes in skeletal muscle and cardiovascular disease.

This Frontiers Research Topic, entitled “Advancements in Therapeutic Strategies for Skeletal Muscle and Cardiovascular Diseases: Integrating Innovative Approaches for Enhanced Outcomes,” consolidates original research and comprehensive review articles that demonstrate how progress in cell and gene therapy is increasingly propelled by a deeper understanding of tissue microenvironments, molecular mechanisms, and pioneering therapeutic platforms. Although the studies target different organs and disease contexts, they share a common objective: to improve tissue repair through the modulation of cellular behavior, immune responses, and therapeutic delivery systems.

Stem cells are characterized by their ability to self-renew and differentiate into various cell types, contingent upon their level of potency (Zakrzewski et al., 2019). In recent decades, stem cells and their derivatives have emerged as promising candidates for regenerative medicine, especially in the treatment of skeletal muscle, cartilage, and cardiovascular diseases, where tissue loss and limited inherent regenerative capacity continue to pose significant therapeutic challenges (Mei et al., 2024; Zakrzewski et al., 2019).

A primary translational obstacle in stem cell-based therapies for myocardial repair is the inefficient migration and homing of transplanted cells to the injured cardiac tissue. In response to this challenge, Hou et al. present an innovative preconditioning approach that utilizes ELABELA (ELA), an endogenous peptide, to augment the migratory capacity of bone marrow–derived mesenchymal stem cells (BMSCs) (Hou et al.). The authors demonstrate that pretreatment with ELA markedly enhances the homing and engraftment of BMSCs within the damaged myocardium, marking the inaugural utilization of ELABELA as a stem cell preconditioning agent in the context of myocardial injury (Hou et al.). This research underscores the potential of endogenous peptides as novel pharmacological tools to overcome critical barriers in regenerative cardiovascular therapy.

Beyond limitations in cell homing, the hostile microenvironment of injured tissues and immune-mediated rejection represent additional major challenges. Alves et al. address these barriers through an innovative strategy that combines cell therapy and gene therapy to modulate the ischemic microenvironment in limb ischemia (Alves et al.). By differentiating genetically edited embryonic stem cells into PHD2-haplodeficient macrophages, the authors generate a pro-resolving immune cell population that promotes tissue repair while attenuating excessive inflammation and immune activation (Alves et al.). This approach constitutes a significant conceptual advance by integrating genetic engineering, immune evasion, and microenvironmental reprogramming to enhance regenerative outcomes.

Immune rejection also poses a substantial obstacle in skeletal muscle regeneration, particularly in Duchenne muscular dystrophy, where the host immune responses limit graft survival following cell transplantation. In this regard, Raiten et al. demonstrate that preconditioning muscle stem cells (MuSC) with hypoxia and interferon-γ (IFN-γ) enhances PD-L1 expression, thereby enabling transplanted cells to evade immune-mediated destruction without the necessity of extensive systemic immunosuppression (Raiten et al.). This study reinforces the potential of preconditioning strategies as a powerful means to improve engraftment and functional recovery in muscle disorders.

Another fundamental challenge in regenerative therapies is the low survival and limited engraftment of transplanted cells within poorly perfused and hostile injury sites. Mitre et al. address this issue using an integrative tissue engineering approach based on 3D muscle-derived stem cell (MDSC) spheroids encapsulated in RGD-modified alginate hydrogels, combined with VEGF preconditioning and paracrine interactions with endothelial cells (Mitre et al.). This strategy significantly enhances cell viability while creating a supportive microenvironment that promotes vascularization and tissue integration, underscoring the potential of biomaterial-based platforms to improve translational success.

While cell encapsulation within biomaterials has become an established strategy to improve stem cell survival and retention, recent advances suggest that nanoencapsulation represents the next frontier in therapeutic delivery. Liao et al. emphasize how nanotechnology facilitates targeted delivery not only of stem cells but also of cell-derived products such as exosomes, bioactive molecules, and conventional drugs (Liao et al.). These methodologies are especially pertinent for poorly vascularized and inherently low-regenerative tissues, such as articular cartilage in osteoarthritis (Liao et al.). By enhancing tissue penetration, retention, and local bioavailability while minimizing systemic toxicity, nano-based platforms broaden therapeutic possibilities beyond cell therapy alone and reinforce nanodrug systems as potent tools in regenerative pharmacology.

The studies in this Research Topic collectively address shared translational challenges, including limited cell survival and engraftment, immune rejection, and hostile tissue microenvironments, through innovative and integrated therapeutic strategies. Spanning diverse disease contexts such as peripheral artery disease, myocardial infarction, Duchenne muscular dystrophy, volumetric muscle loss, and osteoarthritis, these contributions demonstrate that regenerative therapies, despite targeting distinct tissues and employing different stem cell types, are constrained by common biological barriers. Together, this collection highlights convergent design principles that are shaping the next-generation of regenerative pharmacology, advancing the field toward more robust, mechanism-driven, and clinically translatable interventions.

## References

[B1] Mei R. Wan Z. Yang C. Shen X. Wang R. Zhang H. (2024). Advances and clinical challenges of mesenchymal stem cell therapy. Front. Immunol. 15, 1421854. 10.3389/fimmu.2024.1421854 39100671 PMC11294097

[B2] Zakrzewski W. Dobrzyński M. Szymonowicz M. Rybak Z. (2019). Stem cells: past, present, and future. Stem Cell Res. and Ther. 10, 68. 10.1186/s13287-019-1165-5 30808416 PMC6390367

